# Dedifferentiated Liposarcoma With Low-Grade Osteosarcomatous Differentiation: A Rare Case With Long-Term Imaging Follow-Up

**DOI:** 10.7759/cureus.99874

**Published:** 2025-12-22

**Authors:** Kanae Takahashi, Takuya Adachi, Yuki Funauchi, Susumu Kirimura, Ukihide Tateishi

**Affiliations:** 1 Department of Diagnostic Radiology and Nuclear Medicine, Institute of Science Tokyo, Tokyo, JPN; 2 Department of Orthopedic Surgery, Institute of Science Tokyo, Tokyo, JPN; 3 Department of Comprehensive Pathology, Institute of Science Tokyo, Tokyo, JPN

**Keywords:** computed tomography, dedifferentiated liposarcoma, magnetic resonance imaging, orthopaedic oncology, osteosarcomatous differentiation

## Abstract

Dedifferentiated liposarcoma (DDLPS) with low-grade osteosarcomatous differentiation is extremely rare. However, this subtype is considered at a higher risk of early local recurrence than DDLPS without osteogenic differentiation, underscoring the importance of preoperative radiologic diagnosis. Here, we report a case of DDLPS with low-grade osteosarcomatous differentiation in an 80-year-old male located on the left dorsal thigh, in which long-term imaging follow-up was conducted prior to treatment. Gradual enlargement of calcified components in lipomatous masses may indicate the presence of DDLPS with low-grade osteosarcomatous differentiation.

## Introduction

Dedifferentiated liposarcoma (DDLPS), a common form of liposarcoma, is an atypical lipomatous tumor/well-differentiated liposarcoma showing progression to usually non-lipogenic sarcoma (ALT/WDLPS) of variable histological grade [1]. The dedifferentiated areas can exhibit various histological features, and although rare, they can show osteosarcomatous differentiation (reported to account for 0.6% of dedifferentiation cases) [1,2].

In cases with osteosarcomatous differentiation, the histological features generally resemble those of high-grade osteosarcoma; however, low-grade osteosarcomatous differentiation may be observed in rare instances [3-6]. Most ossifications observed in DDLPS are considered neoplastic, and DDLPS with osteogenic differentiation has been reported to have a higher risk of early local recurrence compared with DDLPS without osteogenic differentiation [3]. Therefore, when a lipomatous tumor with calcification is detected on preoperative imaging, considering DDLPS with osteosarcomatous differentiation in the differential diagnosis may help facilitate appropriate therapeutic planning. However, there are only a few case reports on the imaging findings of osteosarcomatous differentiation in DDLPS.

We present a case of DDLPS with low-grade osteosarcomatous differentiation, observed over time on computed tomography (CT) and magnetic resonance imaging (MRI). To our knowledge, no reports have followed the imaging changes over several years in an untreated state.

## Case presentation

**Figure 1:**
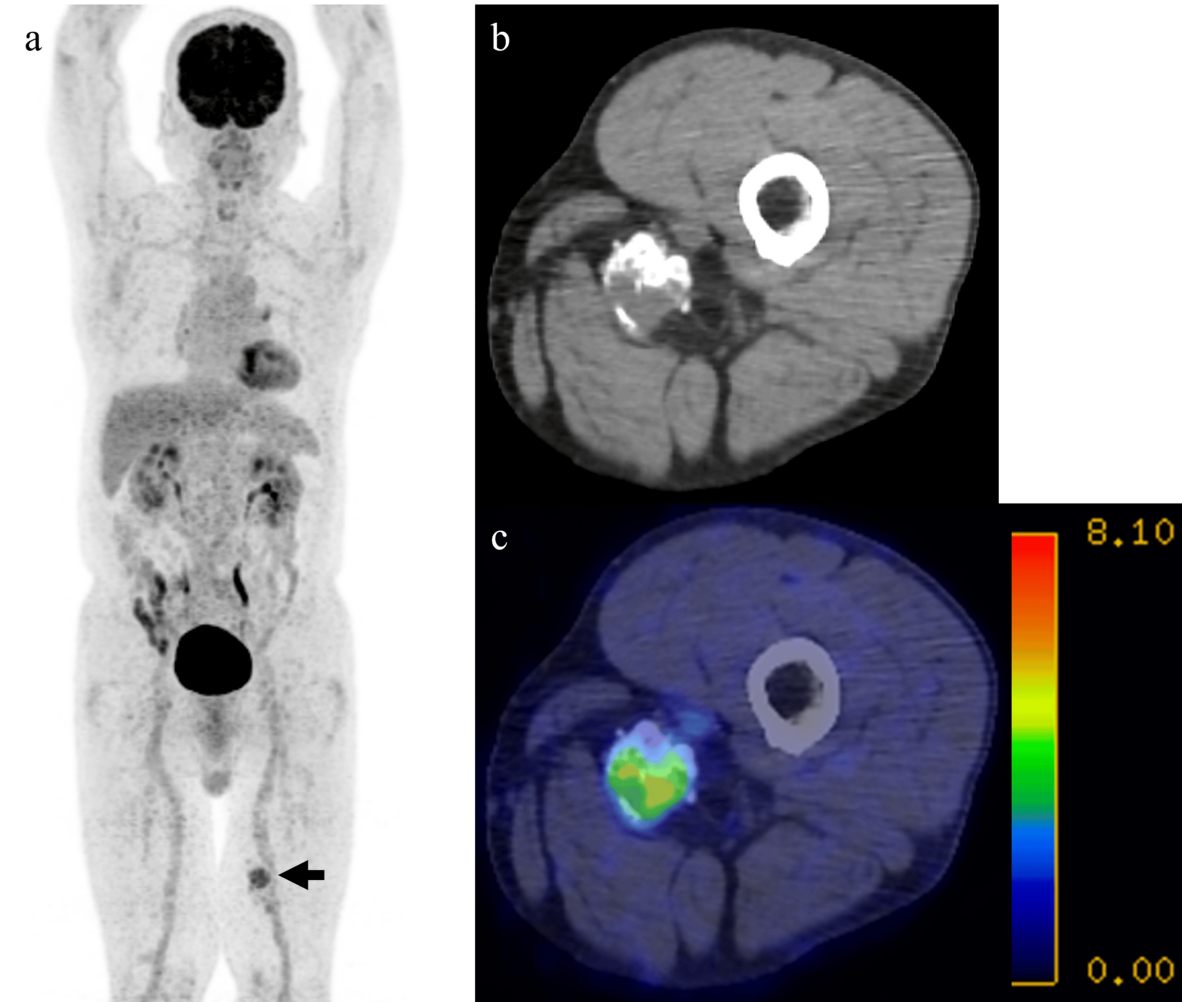
PET/CT images evaluated for suspected primary lung cancer in an asymptomatic 80-year-old male MIP image showing increased FDG uptake in the left thigh (a). Attenuation-correction CT (b) and fused PET/CT image (c), demonstrating a SUV max of 5.6. PET/CT: positron emission tomography/computed tomography, FDG: fluorodeoxyglucose, SUV max: maximum standardized uptake value, MIP: maximum intensity projection

An 80-year-old male, who had a history of lung cancer surgery and was asymptomatic, underwent 18F-fluorodeoxyglucose (FDG) positron emission tomography/CT (PET/CT) at a referring hospital due to suspicion of another primary lung cancer. PET/CT imaging revealed a soft-tissue mass with FDG uptake (maximum standardized uptake value = 5.6) containing calcification and fat in the left dorsal thigh (Figure 1). He was referred to our hospital for evaluation six months later.

**Figure 2:**
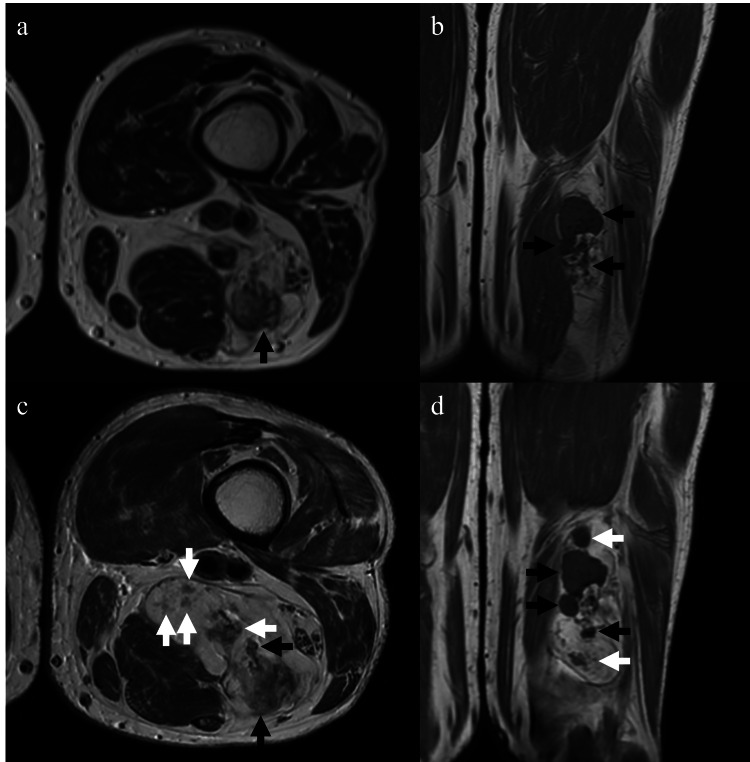
DDLPS with low-grade osteosarcomatous differentiation of the left femur in an 80-year-old male presenting no symptoms Initial MRI (a, b) includes high-intensity areas corresponding with fat and low-intensity areas corresponding with calcification (black arrows) on axial T2-weighted MRI (a) and coronal T1-weighted MRI (b). Follow-up MRI (c, d) reveals noticeable enlargement (black arrows) and new development (white arrows) of the calcified areas, as well as fatty areas. MRI: magnetic resonance imaging, DDLPS: dedifferentiated liposarcoma

Physical examination revealed a large palpable mass in the left thigh, which was not tender to palpation. At the initial visit to our hospital, an MRI revealed a mass measuring 36 × 29 × 81 mm. The lesion exhibited areas of high signal intensity on T1-weighted imaging (T1WI) and T2-weighted imaging (T2WI), corresponding to fatty components, intermixed with areas of low signal intensity on T1WI and T2WI, corresponding to calcification components (Figure 2a-2b).

**Figure 3:**
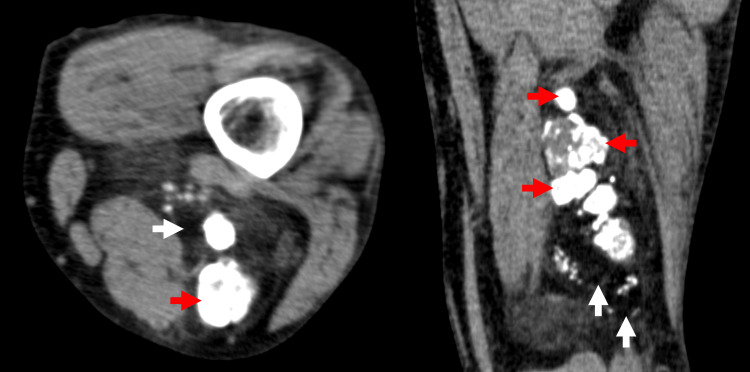
CT images during the CT-guided biopsy Axial (a) and coronal (b) CT images taken during the CT-guided biopsy demonstrate a mass containing both calcified (red arrows) and fatty areas (white arrows). The calcified components have enlarged compared to the previous PET/CT images performed at the referring hospital. PET/CT: positron emission tomography/computed tomography

Combined with the PET/CT findings from the referring hospital, the diagnosis of osteolipoma, ALT/WDLPS with calcification, or venous malformation was suspected. Due to the patient’s age and the prioritization of lung cancer treatment, a watchful waiting approach was selected. Six months later, MRI showed little change in both the non-fatty and fatty components. However, another year later, an MRI scan revealed an overall increase in the size of both the fatty and calcified components. Consequently, a needle biopsy was performed, leading to a pathological diagnosis of DDLPS. The CT images obtained during the CT-guided biopsy (Figure 3) revealed enlargement of the calcified components compared with the previous PET/CT images performed at the referring hospital.

The follow-up MRI, performed two years after the initial scan and just before surgery to assess the status of the lesion, also showed further enlargement and the development of new fatty and calcified components within the lesion (Figure 2c-2d).

Although the slow progression was atypical, the predominant enlargement of the calcified areas and the biopsy-confirmed diagnosis of DDLPS suggested osteosarcomatous differentiation. The preoperative imaging showed no signs of lymph node metastasis or distant metastasis, and a wide resection of the tumor was performed.

**Figure 4:**
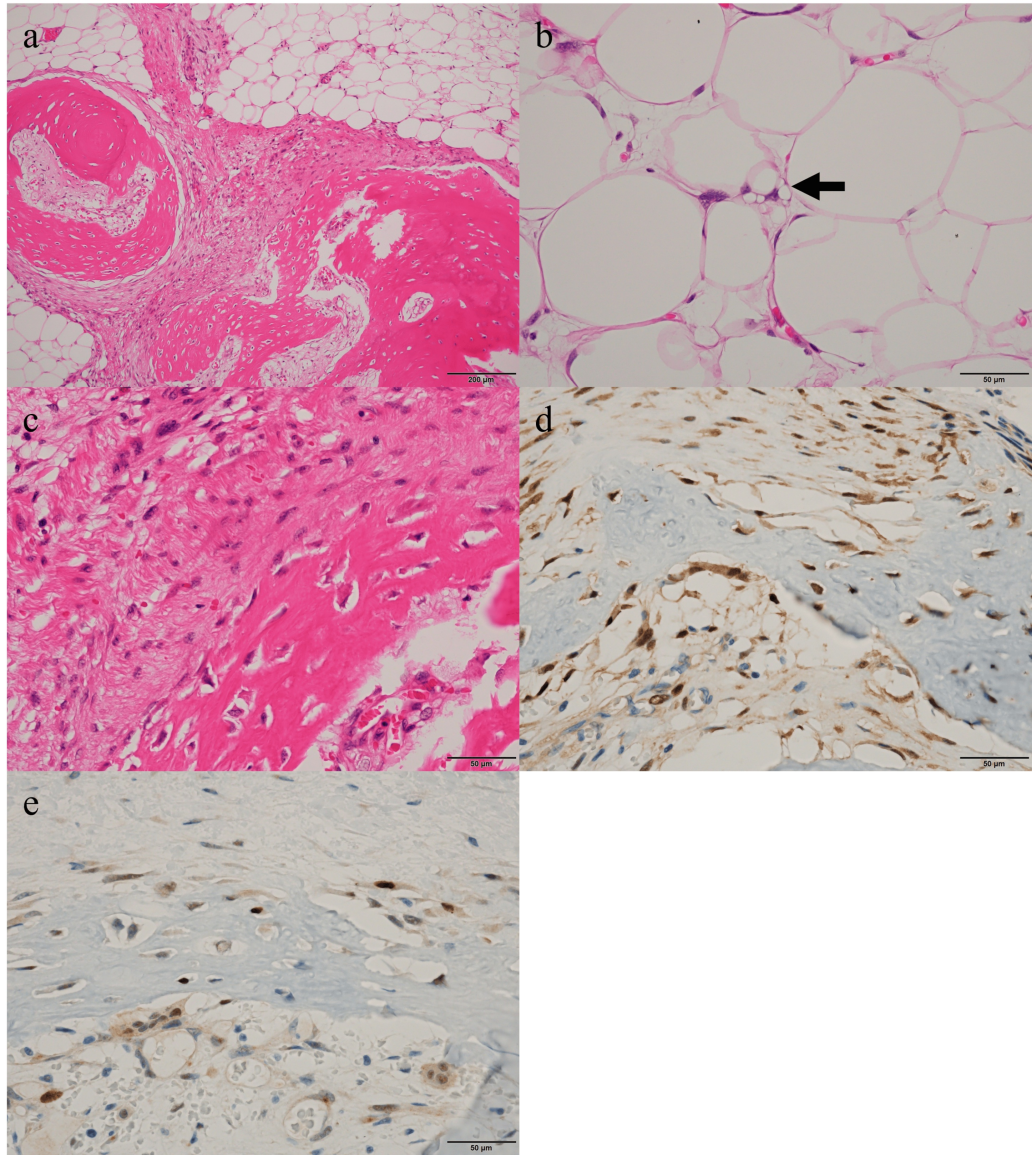
H&E-stained sections and immunohistochemical staining sections (a) The WDLPS and osteosarcoma-like components are intermixed (100×). (b) The lipoblast cells are observed (400×). (c) The spindle-shaped cells form collagen fibers and osteoid or bone in the ossified tissue (400×). (d, e) Both cyclin-dependent kinase 4 (d, 400×) and murine double minute 2 (e, 400×) were positive in osteosarcoma-like components. H&E: hematoxylin and eosin, WDLPS: well-differentiated liposarcoma

The gross specimen was 24 × 9.5 × 5.0 cm in total, with a 4.5 × 4.0 × 3.5 cm yellow, lipomatous mass and 9.0 × 6.0 × 2.5 cm of ossified tissue observed close together, and these components were seen intermixed in some areas. Histologically, the yellowish mass consisted of adipose tissue with varying adipocyte sizes. Enlarged nuclei and multivacuolated lipoblast-like cells were scattered, and cells with bizarre nuclei were also observed (Figure 4a-4b). Additionally, atypical spindle-shaped cells proliferated within the fibrous septa. On the other hand, in the ossified tissue, spindle-shaped cells formed collagen fibers and osteoid or bone tissue against a background of adipose tissue (Figure 4a-4c). Although focal nuclear atypia was present, the overall cytologic atypia of the osteosarcoma-like component was mild, and no marked pleomorphism was identified. The mitotic figures were approximately 1-2 per 10 high-power fields in areas with the highest density of mitoses. Coagulative tumor necrosis was absent. Immunohistochemical staining showed positive results for p16, murine double minute 2, and cyclin-dependent kinase 4 in both areas, with approximately 10% of cells being Ki67 positive in both the lipomatous and osteosarcoma-like components (Figure 4d-4e). The osteosarcoma-like components were intermixed with WDLPS components, resulting in the diagnosis of DDLPS with low-grade osteosarcomatous differentiation (Figure 4a). The French Fédération nationale des centres de lutte contre le cancer score was 2. Although the surgical margins were not confirmed to be negative, the MRI three months after resection showed no signs of local recurrence, and the patient has remained free of recurrence or metastases for four months after resection.

## Discussion

DDLPS with osteosarcomatous differentiation is a lesion in which DDLPS exhibits heterologous differentiation resembling conventional osteosarcoma. The dedifferentiated areas most frequently resemble undifferentiated pleomorphic sarcoma or intermediate-to-high-grade myxofibrosarcoma; however, in 5-10% of cases, they may exhibit osteosarcomatous differentiation [1,2]. In most cases of osteosarcomatous dedifferentiation, the histological appearance resembles high-grade osteosarcoma; however, cases exhibiting abundant mature bone and being histologically indistinguishable from low-grade osteosarcoma have also been reported in recent years [3-5,7]. However, reports of imaging findings are minimal. Furthermore, no reports of cases that underwent imaging follow-up in the untreated stage have been published for several years.

Yamashita et al. reported that ossified DDLPS tends to develop local recurrence earlier than non-ossified DDLPS [3]. Macagno et al. reported a case of WDLPS with low-grade osteosarcomatous differentiation that experienced repeated local recurrences, noting that its low malignant potential may lead to neoplastic adipose components being overlooked during surgery, resulting in incomplete resection and postoperative local recurrence [7]. In this case, careful imaging follow-up is being conducted; however, there has been no local recurrence.

To date, 13 reports have described 14 cases of DDLPS with osteosarcomatous differentiation, including radiological images (Table 1) [2,5,6,8-17]. Among previously reported cases of DDLPS with osteosarcomatous differentiation, only two showed low-grade differentiation. However, more than half of these reports fail to specify the malignancy grade of osteosarcomatous differentiation.

**Table 1:** Cases of DDLPS with osteosarcomatous differentiation with reported radiological findings Ca: calcification, Mets: metastasis, REC: recurrence, M: male, F: female, XR: plain radiography, CT: computed tomography, MRI: magnetic resonance imaging, BS: technetium-99m methyl diphosphonate bone scintigraphy, NA: data not available, NED: no evidence of disease, DOD: died of disease, SCLC: small cell lung cancer, mo: months, DDLPS: dedifferentiated liposarcoma

No.	Author	Year	Age	Sex	Location	Modality	Ca	Fat	Soft tissue components at imaging	Mets	REC	Size (cm)	Grade of the OS change	Postoperative course (mo)
1	Yu et al. [11]	2005	59	F	Left thigh	XR, MRI	+	+	NA	-	-	11	High	NED (NA)
2	Fujii et al. [8]	2013	82	M	Retroperitoneum	CT	+	+	+	-	-	5	High	NED (12mo)
3	Gordhandas et al. [9]	2019	72	M	Retroperitoneum	CT	+	-	+	NA	NA	7	High	NA
4	Wang and Shi et al. [10]	2012	20	F	Neck	CT	+	+	+	-	-	5	High	NED (5mo)
5	Takanami et al. [6]	2005	59	M	Right pleura	CT	+	+	+	-	-	12	Low	NA
6	Zajicek et al. [5]	2017	59	M	Right thigh	XR, CT, MRI	+	+	NA	+	-	NA	Low	Mets (9mo)
7	Toshiyasu et al. [2]	2009	50	M	Retroperitoneum	XR, CT, MRI	+	+	+	+	-	NA	NA	DOD (7mo)
8	Toshiyasu et al. [2]	2009	54	M	Retroperitoneum	XR, CT, BS	+	+	+	-	-	NA	NA	NED (24mo)
9	Zheng et al. [12]	2023	63	M	Rectum	CT	+	-	+	-	-	3	NA	Died of SCLC (18mo)
10	Sun et al. [13]	2012	47	F	Retroperitoneum	CT	+	+	+	-	-	20	NA	NED (12mo)
11	Nguyen et al. [14]	2013	46	M	Retroperitoneum	CT	+	+	+	NA	NA	22	NA	NA
12	Yamamoto et al. [15]	2000	78	M	Left thigh	XR	+	NA	NA	+	-	28	NA	NED (24mo)
13	Sosnowska-Sienkiewicz et al. [16]	2023	60	F	Retroperitoneum	CT	+	+	＋	-	-	5.6	NA	NED (18mo)
14	Anand Rajan et al. [17]	2010	11	F	Posterior mediastinum	CT	+	+	+	-	-	31	NA	NED (NA)

All cases exhibited calcification/ossification within the fatty tissue mass on imaging; similar findings were observed in this case, consistent with previous reports. However, no differences in imaging findings by grade of osteosarcomatous differentiation were identified, indicating that further evidence is needed. Consistent with the concept of low-grade dedifferentiation in DDLPS, the osteosarcomatous component in our case showed only mild cytologic atypia, low mitotic activity, and no tumor necrosis.

In this case and in the report by Zajicek et al. [5], that is, in the few reported cases of DDLPS with low-grade osteosarcomatous differentiation, in which imaging and clinical course were comprehensively documented, the tumors in both cases showed enlargement of non-fatty components, including calcification. However, the rate of increase was slower than the rapid clinical course typically seen in DDLPS. Given the limited number of reported imaging findings, it is challenging to distinguish DDLPS with low-grade osteosarcomatous differentiation from ALT/WDLPS with metaplastic bone formation or osteolipoma on imaging alone. However, when the enlargement of the calcified components is as rapid as, or even faster than, that of the fatty components and does not align with the typically rapid progression seen in DDLPS with high-grade osteosarcomatous differentiation, DDLPS with low-grade osteosarcomatous differentiation, although rare, may be included in the differential diagnosis.

In cases of lipomatous tumors with calcified components, when the growth rate of the calcified components is equal to or faster than that of the fatty components, it may be essential to consider DDLPS with low-grade osteosarcomatous differentiation, which is prone to early local recurrence, and to histologically evaluate both the calcified and fatty components in biopsy specimens.

Furthermore, future studies should assess the correlation between malignancy grade and imaging findings in DDLPS with osteosarcomatous differentiation, as well as their prognosis and local recurrence rates.

## Conclusions

We presented an extremely rare case of DDLPS with low-grade osteosarcomatous differentiation, uniquely documented with longitudinal CT and MRI follow-up over several years in an untreated state. This case demonstrated that despite an atypical, slow growth rate, the progressive enlargement of calcified components in conjunction with fatty components can be a key imaging finding for this entity. Clinicians should therefore consider DDLPS with low-grade osteosarcomatous differentiation when calcified components in lipomatous masses enlarge gradually, as correct preoperative identification is crucial for surgical planning, given the reported higher risk of local recurrence. Further case accumulation is necessary to better characterize the imaging-pathology correlation and prognosis of this rare variant.
